# TgrC1 Has Distinct Functions in *Dictyostelium* Development and Allorecognition

**DOI:** 10.1371/journal.pone.0124270

**Published:** 2015-04-20

**Authors:** Yue Wang, Gad Shaulsky

**Affiliations:** 1 Graduate Program in Structural Computational Biology and Molecular Biophysics, Baylor College of Medicine, Houston, Texas, 77030, United States of America; 2 Department of Molecular and Human Genetics, Baylor College of Medicine, Houston Texas, 77030, United States of America; Université de Genève, SWITZERLAND

## Abstract

The cell adhesion glycoproteins, TgrB1 and TgrC1, are essential for *Dictyostelium* development and allorecognition, but it has been impossible to determine whether their pleiotropic roles are due to one common function or to distinct functions in separate pathways. Mutations in the respective genes, *tgrB1* and *tgrC1*, abrogate both development and allorecognition and the defects cannot be suppressed by activation of the cyclic AMP dependent protein kinase PKA, a central regulator of *Dictyostelium* development. Here we report that mutations in genes outside the known PKA pathway partially suppress the *tgrC1*-null developmental defect. We separated the pleiotropic roles of *tgrC1* by testing the effects of a suppression mutation, *stc^ins^A* under different conditions. *stcA^ins^* modified only the developmental defect of *tgrC1^–^* but not the allorecognition defect, suggesting that the two functions are separable. The suppressor mutant phenotype also revealed that *tgrC1* regulates stalk differentiation in a cell-autonomous manner and spore differentiation in a non-cell-autonomous manner. Moreover, *stcA^ins^* did not modify the developmental defect of *tgrB1^–^*, but the less robust phenotype of *tgrB1^–^* obscures the possible role of *stcA* relative to *tgrB1*.

## Introduction

The social soil amoeba *Dictyostelium discoideum* is used as a model system for the study of development, cell type differentiation and the evolution of multicellularity. During vegetative growth, the amoebae propagate as single cells. Upon starvation, they stop dividing, aggregate into multicellular structures that contain thousands of cells and develop into fruiting bodies composed of two cell types, the reproductive spores and the somatic stalks. The developmental process takes about 24 hours. In the first quarter of development, the cells entrain each other using extracellular cAMP. They then begin to aggregate and after 8–10 hours they form mounds, while starting to differentiate into the two major cell types. At 16 hours, the mound is transformed into a slug, in which the different cell types have sorted out into designated regions along the long axis of the slug – prestalk cells mostly in the front and prespore cells in the back. Eventually, the slug erects itself and the prestalk cells descend through the prespore cell mass as both cell types undergo terminal differentiation into stalks and spores.

TgrC1 has several important functions in development. TgrC1 is a single-pass transmembrane glycoprotein with a large extracellular domain and a short cytoplasmic tail. It was first identified as an EDTA-insensitive cell-cell adhesion molecule [1,2]. Adhesion is a critical component in multicellularity because it holds the cells together and often participates in signaling. TgrC1 expression starts at the mound stage and peaks at the slug stage [1]. It accounts for about 50% of the total adhesion at the slug stage [3] and its adhesion partner is TgrB1, another single-pass transmembrane glycoprotein [4]. Mutants lacking *tgrC1* function are developmentally arrested at the loose aggregate stage and exhibit defects in differentiation of both cell types, suggesting that TgrC1 has a role in controlling differentiation [5]. Self-recognition is an important social feature of *Dictyostelium*. When strains with different genetic backgrounds develop together, the cells tend to sort out in aggregates and undergo further development in clonal aggregates [6]. TgrB1 and TgrC1 are necessary and sufficient to exclusively mediate this process. When two strains have matching TgrB1/TgrC1, they aggregate and co-develop regardless of their genetic backgrounds. Similarly, when two strains have non-matching TgrB1/TgrC1, they sort out and develop clonally even if the rest of the genome is identical [7]. TgrC1 and TgrB1 participate in adhesion and in signaling, both in development and in social recognition, but it is not clear whether the functions are overlapping or distinct.

We used a genetic screen for suppressors of the *tgrC1* ^*-*^ phenotype to begin to address that question. We surmised that such suppressors would uncover genetic components in specific pathways. If such suppressors would modify only one of the phenotypes but not another, or if they modify only the *tgrC1* ^*-*^ phenotype but not the *tgrB1* ^*-*^ phenotype, then the functions must be distinct. We could use such suppressors as probes to dissect the multiple functions of TgrC1 under different conditions, and such suppressors may also provide an opportunity to differentiate the functions of TgrB1 and TgrC1 in development. We generated random mutations in a *tgrC1* ^*-*^ strain and selected for strains that carried a second-site mutation and were able to sporulate. We identified three suppressors of *tgrC1* that partially restored the function in development and thus in cell-type differentiation. We further characterized one of the suppressors, *stcA* ^*ins*^, and tested its effect on self recognition and on cell-type differentiation to reveal the regulatory functions of TgrC1. The results led us to propose that TgrC1 utilizes different pathways to mediate differentiation and self recognition, and that the self recognition function is separable from the differentiation function. Moreover, TgrC1 regulates the differentiation of the two cell types differently. It regulates stalk formation in a cell-autonomous way and sporulation in a non-cell-autonomous way. We also found that TgrC1 and TgrB1 have distinct roles in signal transduction. We propose that TgrC1 participates in a critical control point in *Dictyostelium* development, allowing the cells to integrate inputs from both differentiation signals and from self recognition signals and to mount the proper responses.

## Results

### Suppressor mutations partially rescue the developmental defect of tgrC1^-^

We used restriction enzyme mediated insertion (REMI) to perform a screen for suppressors of the *tgrC1* ^*-*^ phenotype. During development in a pure population, *tgrC1* ^*-*^ is arrested at the loose aggregate stage. It does not form fruiting bodies and produces very few spores [5]. We therefore selected for suppressor mutations that produced viable spores that could germinate, grow in association with bacteria and develop into fruiting bodies. We screened a total of 8,000 insertion mutants and identified three independent suppressor strains in which we identified three genes that have not been characterized before. We named them *stcA* (DDB_G0267562), *stcB* (DDB_G0270410) and *stcC* (DDB_G0274171), where stc stands for suppressor of *t* *gr* *C* *1* ^*-*^. The screen was not carried to saturation. To make sure that the suppression was due to the mutations we discovered, we recapitulated the mutations in a fresh *tgrC1* ^*-*^ background and observed the same suppression phenotype (S1 Fig and data not shown).

**Fig 1:**
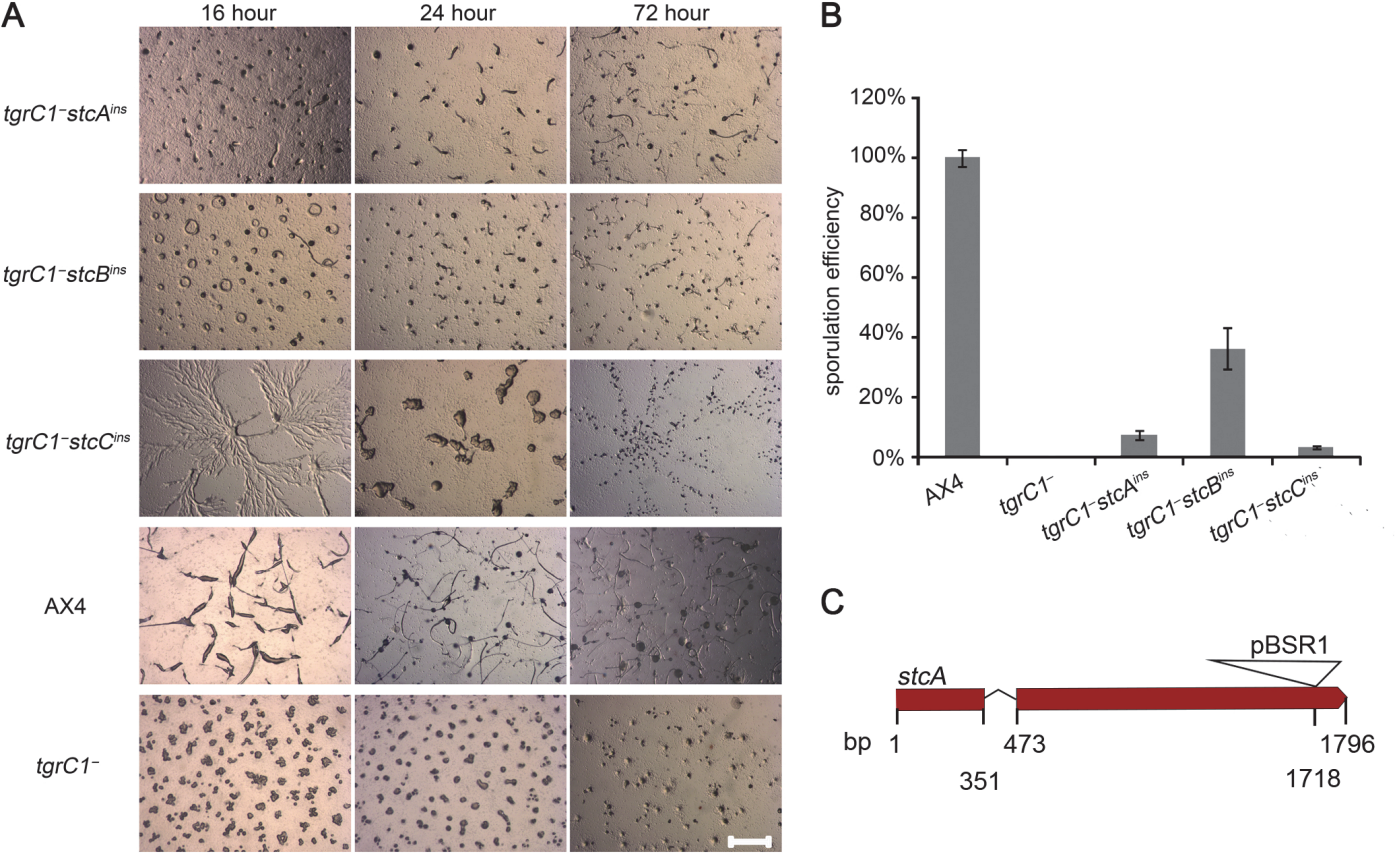
*tgrC1^−^* development is partially restored by suppressor mutations. A. We developed the suppressor strains (*tgrC1^−^stcA^ins^*, *tgrC1^−^stcB^ins^* and *tgrC1^−^stcC^ins^*), the laboratory wild-type strain AX4 and the parental *tgrC1^−^* strain on non-nutrient agar. We photographed the cells with light microscopy from above at 16 hour, 24 hour and 72 hour as indicated (strain genotypes are indicated on the left). Scale bar, 1 mm. B. We determined the sporulation efficiency of the same strains at 72 hour of development on black filters. The results are shown as the average (n = 3) and s.d. of the fraction of cells that made spores normalized to the wild type AX4 (%, y-axis). Strain genotypes are indicated on the bottom. C. An illustration of the *stcA* gene and the insertional mutation. The thick red line represents the two exons and the thin angled lines represent the single intron. The triangle represents the pBSR1 plasmid, which was inserted at the 3’-end of the gene. The inserted plasmid is not drawn to scale. Numbers (bp) below the lines indicate the beginning and the end of the gene model, the predicted splicing borders and the insertion site.

We developed the three suppressor strains and compared their development on non-nutrient agar with those of the laboratory wild type, AX4, and the parental *tgrC1* ^*-*^ cells (Fig 1A). All three suppressor strains developed past the loose aggregate stage, but they all had developmental defects compared with the wild type. After the loose aggregate stage, the aggregate size of all strains was small compared to AX4. In addition, they all formed fruiting bodies eventually, but none of them formed fruiting bodies at 24h and their development was rather unsynchronized. We quantified the sporulation efficiency of these strains and found that in all three cases it was significantly higher than the parental *tgrC1* ^*-*^ strain but lower than that of the wild type AX4 cells (Fig 1B). In summary, the three mutations we discovered were able to partially suppress the developmental defect of *tgrC1* ^*-*^, as indicated by advanced morphology and improved sporulation. We further characterized the effects of the mutation in the *stcA* gene, which encodes a putative protein of 557 amino acids without any distinctive features. The insertion site in *stcA* occurred at base pair 1718, toward the 3’ end of the gene (Fig 1C). We tested the expression of the disrupted *stcA* mRNA by RT-PCR and found that the region upstream of the insertion site was expressed at levels comparable or slightly higher than the wild type and the parental *tgrC1* ^*-*^ strain (S2A Fig). The region overlapping the insertion site was equally abundant in the wild type and the parental *tgrC1* ^*-*^ strain, but no RT-PCR product was observed in the *tgrC1* ^*-*^ *stcA* ^*ins*^ mutant, validating that this region was disrupted by the insertion (S2B Fig). These results indicate that the insertion disrupted the gene and the resulting mRNA, suggesting that the protein may have been disrupted as well.

### *tgrC1* ^*-*^ has a cell-autonomous defect in spore differentiation, and a non-cell-autonomous defect in stalk differentiation

**Fig 2:**
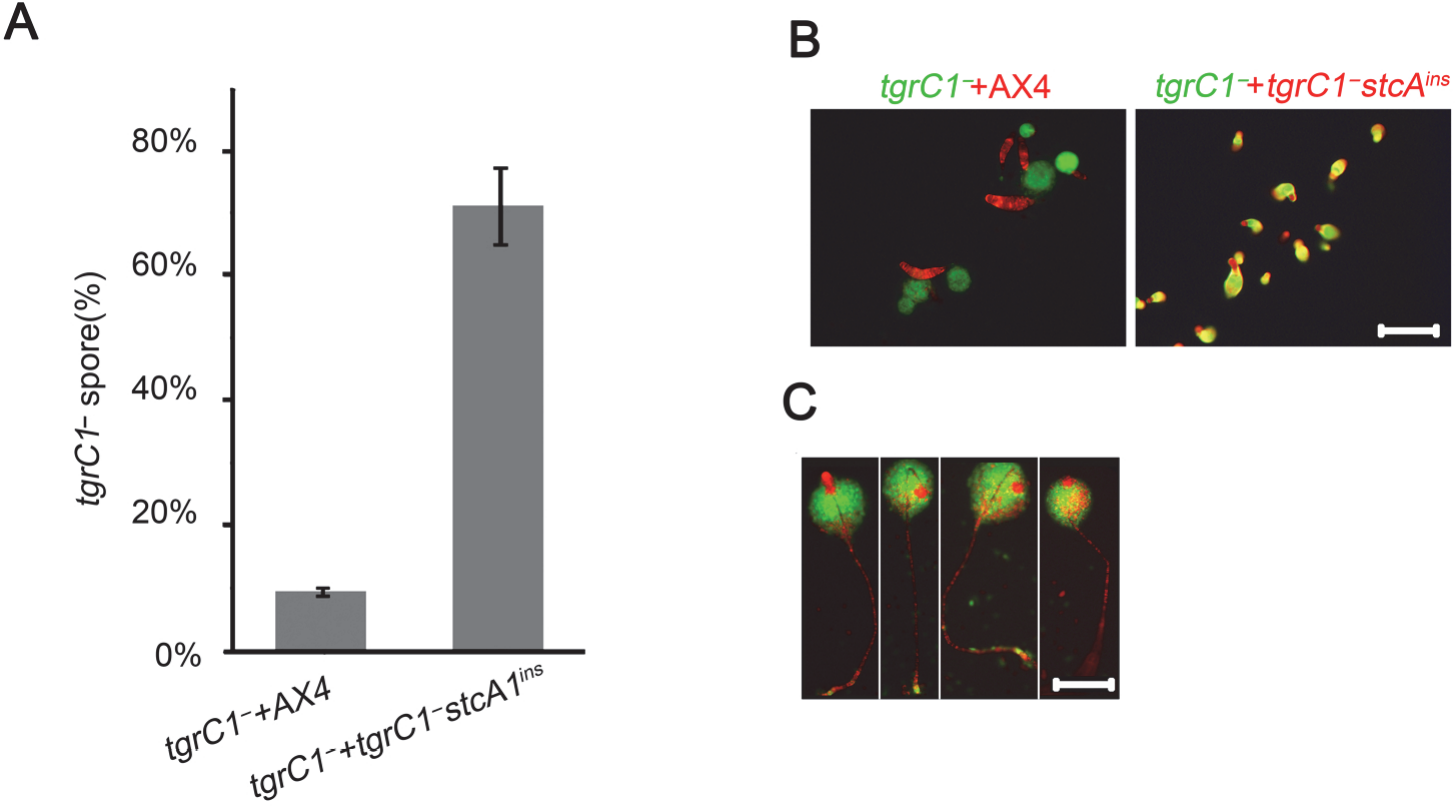
The *tgrC1* ^*-*^ sporulation defect is non-cell autonomous and the stalk development defect is cell-autonomous. A. We mixed *tgrC1^−^* with *tgrC1^−^stcAins* or AX4, as indicated, at 1:1 ratios, and developed the mixture for 72 hours. We collected the spores, germinated them in association with *Klebsiella aerogenes* to allow single plaque formation and recorded the fraction (%) of *tgrC1^−^* plaques relative to the total number of plaques. The average fraction and the s.d. are shown (y-axis; n = 3). The overall sporulation efficiency of the *tgrC1^−^* and *tgrC1^−^stcAins* strains was similar to that of *tgrC1^−^stcAins* alone (Fig 1B). The overall sporulation efficiency of the mix between *tgrC1^−^* and AX4 was about half of that of AX4 alone (Fig 1B). We mixed GFP-labeled *tgrC1^−^* (green text) with RFP-labeled AX4 or *tgrC1^−^stcAins* (red text) and developed the mixture of cells on non-nutrient agar. We photographed the structures at 16 hour (B) and single fruiting bodies of GFP-labeled *tgrC1^−^* and RFP-labeled *tgrC1^−^stcAins* at 48 hour (C). Scale bar, 0.2 mm.

The *tgrC1* ^*-*^ mutation affects several developmental processes, including aggregation and cell-type differentiation and some of these effects are non-cell autonomous [5,7–9]. In order to examine the mode of suppression in the *tgrC1* ^*-*^ *stcA* ^*ins*^ strain, we mixed equal proportions of *tgrC1* ^*-*^ with AX4 or with *tgrC1* ^*-*^ *stcA* ^*ins*^, developed them and determined the proportions of the strains in the resulting spores (Fig 2A). When *tgrC1* ^*-*^ cells were mixed with the wild type AX4, about 10% of the spores in the fruiting bodies had the *tgrC1* ^*-*^ genotype, consistent with previous findings that the *tgrC1* ^*-*^ phenotype has a weak non-cell autonomous property. In the mix of *tgrC1* ^*-*^ and *tgrC1* ^*-*^ *stcA* ^*ins*^ we found about 70% *tgrC1* ^*-*^ spores, suggesting that the *stcA* ^*ins*^ mutation enhances sporulation in a non-cell-autonomous way in both *tgrC1* ^*-*^ and *tgrC1* ^*-*^ *stcA* ^*ins*^. One possibility is that *tgrC1* ^*-*^ formed more spores than its fair share in the chimera by avoiding contribution to stalks. To examine that possibility we labeled the strains separately with either Green- or Red-fluorescence proteins. We mixed the cells at equal proportions, developed them, and tracked their location in the mixed aggregates during development. At 16 hours of development, we observed that the wild-type AX4 cells formed slugs and left the *tgrC1* ^*-*^ aggregates behind, while the *tgrC1* ^*-*^ *stcA* ^*ins*^ cells remained mixed with *tgrC1* ^*-*^ and formed chimeric slugs. Interestingly, the cells were not evenly mixed in the chimeric slugs—the prestalk region in the slug anterior contained predominantly *tgrC1* ^*-*^ *stcA* ^*ins*^ cells (Fig 2B). Following development of the *tgrC1* ^*-*^ *stcA* ^*ins*^ and *tgrC1* ^*-*^ chimerae to fruiting body formation revealed that the stalks were predominately composed of *tgrC1* ^*-*^ *stcA* ^*ins*^ cells and the spore-containing sori consisted of mostly *tgrC1* ^*-*^ cells (Fig 2C). These observations suggest that *tgrC1* ^*-*^ formed more spores than its fair share due to its inability to form stalks. This was not the case in the mixes with the wild type, because the wild type segregated from the *tgrC1* ^*-*^ cells. Therefore, the inability of *tgrC1* ^*-*^ *stcA* ^*ins*^ to segregate from *tgrC1* ^*-*^ contributed to the increased proportion of *tgrC1* ^*-*^ in the spores. In addition, the absence of *tgrC1* ^*-*^ from the stalks of the chimeric fruiting bodies suggests that the stalk-differentiation defect of *tgrC1* ^*-*^ is cell-autonomous.

### *stcA* ^*ins*^ does not modify the phenotype of *tgrB1* ^*-*^

**Fig 3:**
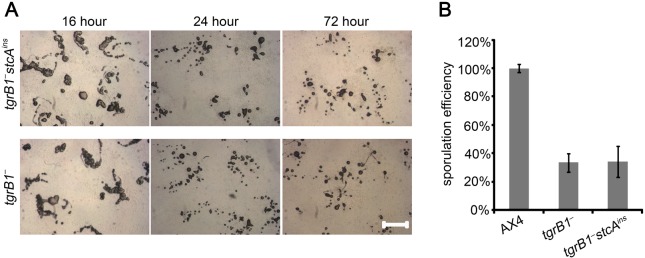
*stcA^ins^* does not modify the *tgrB1^−^* phenotype. A. We developed *tgrB1^−^* and *tgrB1^−^stcA^ins^* on non-nutrient agar and photographed the structures from above with light microscopy at 16 hour, 24 hour and 72 hour as indicated on the top. Strain genotypes are indicated on the left. Scale bar, 0.2 mm. B. We measured the sporulation efficiency of *tgrB1^−^* and *tgrB1^−^stcA^ins^* at 72 hour. The results are shown as the average and s.d. of the fraction of cells (%, y-axis; n = 3) that made spores scaled to the wild type AX4. Strain genotypes are indicated on the bottom.

The observation that *tgrC1* and *tgrB1* function in a common signaling pathway [4,7,8] prompted us to test whether the *stcA* ^*ins*^ mutation could suppress the *tgrB1* ^*-*^ phenotype as well. We generated the *stcA* ^*ins*^ mutation in *tgrB1* ^*-*^ cells and followed the developmental morphology and sporulation efficiency of the resulting *tgrB1* ^*-*^ *stcA* ^*ins*^ strain in comparison with the parental *tgrB1* ^*-*^ strain and the wild type, AX4. We observed no significant difference between the morphologies of *tgrB1* ^*-*^ *stcA* ^*ins*^ and *tgrB1* ^*-*^ strains at any time point (Fig 3A). We also found no significant difference in the sporulation efficiency (Fig 3B). The different behavior of the *stcA* ^*ins*^ mutation in the *tgrB1* ^*-*^ background, compared to the *tgrC1* ^*-*^ background, suggests that *stcA* has a limited effect on the TgrB1-controlled developmental pathway.

### *stcA* ^*ins*^ does not modify the kin-recognition function of *tgrC1* ^*-*^

**Fig 4:**
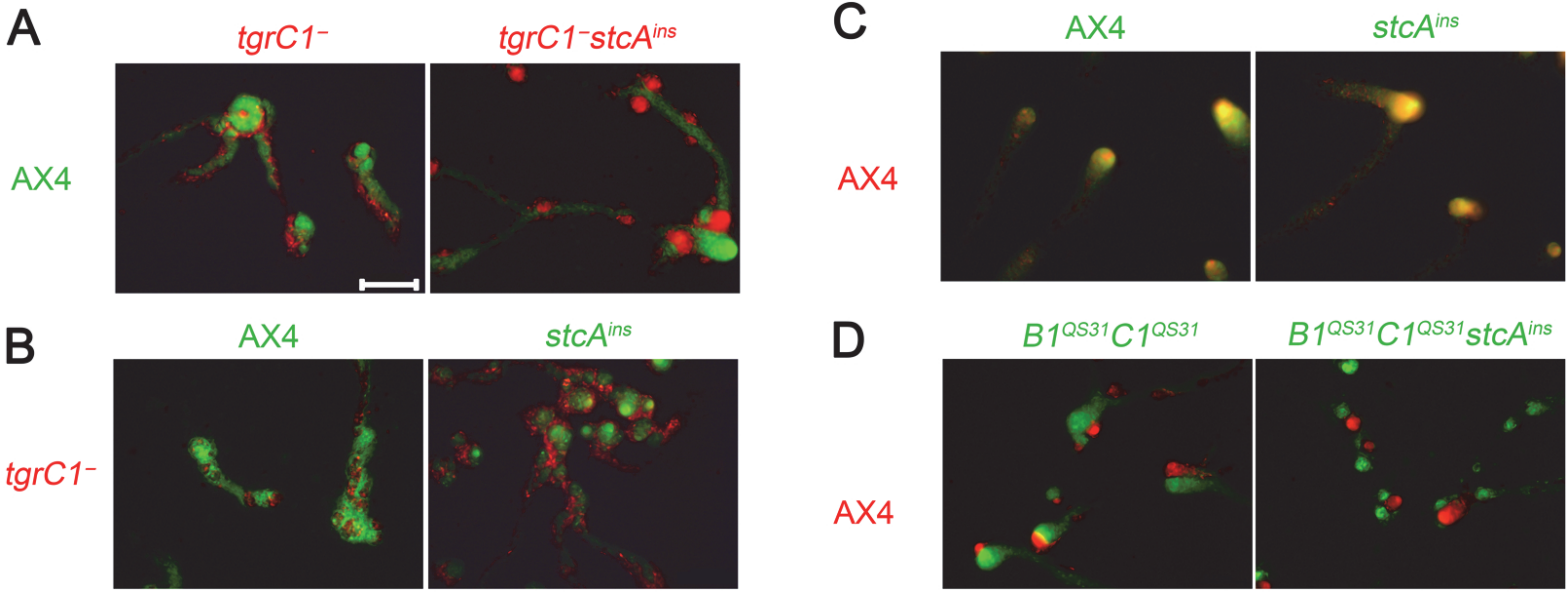
*stcA^ins^* does not modify kin recognition. We labeled cells with GFP (green text) or RFP (red text), grew them independently, mixed two strains at 1:1 ratios and allowed them to develop together on non-nutrient agar. Photographs were taken at 10 hour of development. Cells with the genotypes indicated on the left side of each row were mixed with cells of different genotypes as indicated above each panel. Scale bar, 0.2 mm.

In addition to a critical role in development, *tgrC1* has a role in self recognition [7,8]. It was therefore interesting to test whether the *stcA* ^*ins*^ mutation had an effect on that role of *tgrC1* as well. We generated the *stcA* ^*ins*^ mutation in an otherwise wild type background and also in the double gene replacement strain *tgrB1* ^*QS31*^ *tgrC1* ^*QS31*^, which segregates from the parental AX4 strain due to the different *tgrB1-tgrC1* allotype [7,8]. We labeled the strains with either GFP or RFP and mixed them in equal proportions with several test strains to assess self recognition. In Fig 4A, *stcA* ^*ins*^ was recapitulated in the *tgrC1* ^*-*^ background, and the resulting double mutant *tgrC1* ^*-*^ *stcA* ^*ins*^ behaved like *tgrC1* ^*-*^ in chimerae with the wild type – both strains segregated from the wild type, suggesting that the *stcA* ^*ins*^ mutation does not suppress the self-recognition defect conferred by the *tgrC1* ^*-*^ mutation. Similarly, when *stcA* ^*ins*^ was recapitulated in the AX4 background, the resulting mutant segregated from *tgrC1* ^*-*^ (Fig 4B), but associated with AX4 (Fig 4C), a behavior that was indistinguishable from that of the wild type, AX4. We also recapitulated *stcA* ^*ins*^ in tgr*B1* ^*QS31*^ *tgrC1* ^*QS31*^. The resulting strain was indistinguishable from the parental tgr*B1* ^*QS31*^ *tgrC1* ^*QS31*^, segregating from AX4 in chimerae (Fig 4D). These experiments indicate that the developmental function of *tgrC1* is separable from its function in kin recognition, suggesting that *tgrC1* has distinct functions in development and in kin recognition.

## Discussion

The cell-cell adhesion glycoprotein TgrC1 [10] is essential for development [5,9] and for kin recognition [7,8]. Inactivation of the *tgrC1* gene causes a developmental arrest at the loose aggregate stage and a subsequent inability to differentiate prespore and prestalk cells [5]. Many developmental defects of this type can be suppressed by constitutive activation of PKA. For example, defects in *tagC* and *dhkA* cause developmental arrests at different developmental stages, but both can be suppressed by activation of PKA [11]. Surprisingly, the defect of *tgrC1* ^*-*^ cannot be suppressed by constitutive expression of PKA [12], suggesting that TgrC1 is involved in a parallel signaling pathway. Our genetic screen identified three suppressor mutations, all of which partially restore the ability of *tgrC1* ^*-*^ cells to form fruiting bodies and spores. The suppressor genes have not been identified before, suggesting that they are not directly involved in the well-characterized PKA pathway. These findings support the idea that TgrC1 is involved in a signaling pathway independent of the central PKA pathway in *Dictyostelium* development.

TgrC1 has a role in cell-cell communications during development [9]. This finding focused our interest on the *stcA* ^*ins*^ mutation because it distinguished the regulatory roles of *tgrC1* in stalk and spore differentiation. Specifically, mixing *tgrC1* ^*-*^ cells with AX4 (wild type) cells resulted in the production of very few *tgrC1* ^*-*^ spores but mixing *tgrC1* ^*-*^ cells with the suppressor strain *tgrC1* ^*-*^ *stcA* ^*ins*^ resulted in a majority of *tgrC1* ^*-*^ spores. We also observed that *tgrC1* ^*-*^ cells segregate from AX4 cells but not from the suppressor *tgrC1* ^*—*^ *stcA* ^*ins*^. Based on these findings, we propose that *tgrC1* ^*-*^ cells have a non-cell autonomous defect in spore production, probably because they are unable to produce an extracellular signal necessary for that process. We propose that both AX4 and *tgrC1* ^*-*^ *stcA* ^*ins*^ cells have the ability to provide *tgrC1* ^*-*^ cells with the missing signal, but the segregation of AX4 from the *tgrC1* ^*-*^ cells limits the access of the *tgrC1* ^*-*^ cells to the signal. Our findings also suggest that *tgrC1* participates in stalk differentiation in a cell-autonomous manner because the *tgrC1* ^*-*^ cells did not participate in the stalk in any of the mixing experiments. This observation is consistent with the finding that *tgrC1* mRNA is preferentially expressed in prestalk cells at late stages of development [9]. However, the suppression of *tgrC1* ^*-*^ by *stcA* ^*ins*^ is relatively weak, suggesting that other genetic components are involved in controlling differentiation and development. We have not captured these components in our genetic screen either because we did not carry the screen to near saturation or because the insertional mutagenesis method we used cannot reveal these genes.

Mutations that have different effects on cell-type differentiation have been reported before. For example, prestalk-specific expression of *pkaRm*, a dominant negative regulator of PKA activity, results in a cell-autonomous defect in stalk formation and a non-cell autonomous defect in sporulation [13]. Similarly, inactivation of the prestalk specific gene *tagB* results in a cell-autonomous defect in stalk formation and a non-cell autonomous defect in sporulation [14]. Subsequent studies have shown that prespore and prestalk cells are engaged in an elaborate signaling cascade during terminal differentiation [15]. Both *pkaRm* and *tagB* affect the PKA signaling cascade that regulates terminal differentiation. Since activation of PKA does not suppress the *tgrC1* ^*-*^ defect, it is quite possible that *tgrC1* and *stcA* regulate terminal differentiation in a PKA-independent manner.

Since *tgrC1* is involved in multiple functions, the phenotype of *tgrC1* ^*-*^ at any given time in development is a sum over all its current and previous functions. We would need to integrate the separate functions of adhesion, developmental control and kin-recognition to account for the cells’ behaviors during development, as evidenced by the *tgrC1* ^*-*^ spore proportion difference between the chimeric development of *tgrC1* ^*-*^ with AX4 and that with *tgrC1* ^*-*^ *stcA* ^*ins*^. Due to segregation, there is only a small fraction of *tgrC1* ^*-*^ cells in the AX4 chimeric aggregates whereas there are 50% of *tgrC1* ^*-*^ cells in the *tgrC1* ^*-*^ *stcA* ^*ins*^ chimeric aggregates, partly accounting for the significant difference of the final *tgrC1* ^*-*^ spore proportion. In this report we have shown that stalk differentiation of *tgrC1* ^*-*^ is defective in a cell-autonomous way. However previous findings have demonstrated that stalk differentiation is partially regulated by the extracellular signaling molecule DIF-1 [16,17]. Others have shown that some *tgrC1* ^*-*^ cells could be observed in the stalks of chimeric fruiting bodies of *tgrC1* ^*-*^ and AX4 [9]. These findings support a non-cell-autonomous regulation of stalk differentiation. We argue that since AX4 segregates from *tgrC1* ^*-*^, the abundance of *tgrC1* ^*-*^ cells in either stalks or sori in the final fruiting bodies is low, as indicated by the small number of spores of *tgrC1* ^*-*^ observed in the final spore mass [9]. Therefore, the overall rescue of *tgrC1* ^*-*^ stalk differentiation in the chimerae is limited, suggesting that the non-cell-autonomous regulation of stalk differentiation is weak. In addition, differential adhesion and sorting are likely components of cell-type differentiation and morphogenesis. In mixtures with AX4 cells, the *tgrC1* ^*-*^ cells are likely carried into the aggregates due to incomplete segregation, whereas in mixtures with *tgrC1* ^*-*^ *stcA* ^*ins*^, the *tgrC1* ^*-*^ cells are included in the aggregate but excluded from the prestalk region. It is likely that the different segregation patterns in the different contexts subject the cells to different signals and hence to different fates in subsequent stages of development. The fact that most of the *tgrC1* ^*-*^ cells are absent from the stalks without segregation, and the finding that *tgrC1* ^*-*^ *stcA* ^*ins*^ cells were able to make proper stalks suggest that *tgrC1* ^*-*^ has a direct role in stalk differentiation.

PKA is a central regulator of *Dictyostelium* development and cAMP signals are executed through PKA activation at every stage of development, as demonstrated by the finding that over-expression of PKA-C bypasses the need for cAMP throughout development [18]. In this report, we propose that *tgrC1* acts as another important regulator of *Dictyostelium* development. Unlike the ubiquitous role of PKA throughout development, the findings in this report suggest that *tgrC1* integrates important functions such as differentiation, adhesion and kin recognition, into a critical time at which the transition from unicellularity to multicellularity takes place and cell-type differentiation initiates.

One of the most important findings we made is that the role of *tgrC1* in kin recognition is separable from its role in development. We provided further evidence of the separation of function by showing that the *stcA* ^*ins*^ mutation did not modify the segregation behaviors of other related strains. Moreover, *stcA* ^*ins*^ did not affect the phenotypes of other mutants, suggesting that *tgrC1* may have a separate role in kin recognition that is not entirely overlapping with its role in differentiation. Although the function of *stcA* is not known yet and its sequence does not provide clues in that regard, the mutation and its phenotype are still very informative with respect to the functions of *tgrC1*, which was the goal of this study.

The TgrB1 and TgrC1 proteins may work as a pair in both development and kin recognition [7,8]. We have shown that a suppressor of *tgrC1* ^*-*^ does not alter the phenotype of *tgrB1* ^*-*^. The result is inconclusive because *tgrB1* ^*-*^ phenotype is less robust than the *tgrC1* ^*-*^ phenotype in terms of both sporulation efficiency and developmental morphology. The insignificant difference of sporulation efficiency between *tgrB1* ^*-*^ and *tgrB1* ^*-*^ *stcA* ^*ins*^ could be due to weak suppression compounded with the variability of the *tgrB1* ^*-*^ phenotype, or due to the lack of suppression at all.

## Materials and Methods

### Cell growth, development and transformation

We grew the cells in shaking suspension in HL-5 medium, supplemented with 10μg/mL G418, 10μg/mL Blasticidin S or 20μg/mL uracil, as necessary. We allowed the cells to grow for 24 hours without drugs before the experiments. We harvested the cells at the logarithmic growth phase and developed them on nitrocellulose filters [19] or on non-nutrient agar [20] as previously described. We carried out plasmid transformations as described [6].

### Mutagenesis, selection and identification of the mutated genes

We performed restriction enzyme mediated insertion (REMI) as described [21]. We collected and pooled Blasticidin S resistant cells from four transformations after 6 days of drug selection, spread 5 x 10^5^ cells on each 10 cm SM agar plates in association with *Klebsiella aerogenes*. Immediately after the amoebae cleared the bacterial lawns, we harvested the cells and vortexed for 1 min in 20 mM potassium phosphate buffer (pH 6.2) with 0.1% NP40 to select for spores. We plated about 100 spores on each 10 cm SM agar plate in association with *Klebsiella aerogenes* to allow clonal germination, growth and development. We identified single plaques that contained any fruiting bodies as suppressor mutants of *tgrC1* ^*-*^. We performed plasmid rescue as described to identify the mutated genes [22]. We cloned *stcA* with *Spe*I, *stcB* with *Cla*I, and *stcC* with *Eco*RI. We identified the genes by sequencing the insertion sites with SP6 and T7 primers.

### Nucleic acid manipulation

We prepared genomic DNA as described [23] and performed Southern blot analysis as described [24]. We made the Southern blot probes by PCR. We used the pBSR1 plasmid [21] as the template for Blasticidin S resistance (BSR) cassette and AX4 genomic DNA for all the gene probes. BSR cassette probe primers: 5’-AGTAGAAGTAGCGACAGAGAAGA-3’, 5’-TGGCTGTTTTACATCTAATGC-3’; *stcA* probe primers: 5’-CTTATTGTCTTGTTTCATGTCCTGG-3’, 5’-caatatgaccattttcttcaatgtg-3’; *stcB* probe primers: 5’- GGTGGTTTTTGATGTTGATG-3’, 5’-CATTATTATCATCCTCTTCATCAC-3’; *stcC* probe primers: 5’-CATTGAATTGCACTGCATTCTC-3’, 5’-GAACCCCACTAATTTAAATTTACTGC-3’. We cloned the insertion sits as described [21].

### Construction of new strains and labeling with fluorescence

We used the rescue plasmids to recapitulate the insertional mutations in different backgrounds. We used the pA15/tdTomato and pDXA-GFP2 [8] to label strains with RFP and GFP respectively.

### Segregation assay

We performed segregation assays as described [8]. Briefly, we grew the cells in pure populations, harvested them, mixed two strains at 1:1 ratio and developed them on non-nutrient agar. We photographed the developmental structures with fluorescence microscopy and merged the images without further manipulation.

### Sporulation efficiency

We grew and developed the cells as indicated above. We collected the spores after 72 hours by vortexing the entire filters for 1 min in 20 mM potassium phosphate buffer (pH 6.2) with 0.1% NP40. We then counted the spores. Sporulation efficiency was calculated as the fraction (percent) of spores relative to the number of cells at the start of development. The sporulation efficiency test of each strain contained three independent biological replicates. Each biological replicate included three technical replicates. We averaged (mean) the sporulation efficiencies of the three technical replicates of each strain for every biological replicate, and then normalized the efficiency of every strain to wild type AX4, which was performed in parallel. The final sporulation efficiency was calculated by averaging the data from the three biological replicates.

### RNA extraction and reverse transcription PCR

We performed the experiments as described [8]. We collected RNAs at 20 hours of development for all strains tested. The primer set we used to amplify the *stcA* fragment upstream the insertion site were: 5’-CCACCACCATTATCAACTAG-3’, 5’-GATCATAAAAACCAGGACATG-3’. The primer set we used to amplify the fragment spanning the insertion site were: 5’-CCACCACCATTATCAACTAG-3’, 5’-TTACTTAAATGCATTAAAATCAA-3’.

## Supporting Information

S1 Fig Southern blot analysis. We prepared genomic DNAs from all the strains tested (relevant genotypes are indicated above the lanes; ‘suppressor’ represents the original suppressor strain), digested the DNA samples with SpeI, and separated the fragments on 0.8% agarose gel. After transferring the DNAs to nitrocellulous membranes, we processed the blots with probes against *stcA* or against the blasticidin S resistance cassette (BSR) as indicated below the lanes. MW – molecular weight markers (Kb). Arrows point to the wild type (WT) and mutant (ins) *stcA* bands. The SpeI fragment of *stcA* gene is about 2.2 Kb, and the pBSR1 insertion size is approximately 4.1 Kb.
(TIF)

S2 Fig *stcA* mRNA expression. We developed the strains *tgrC1* ^*-*^ *stcA* ^*ins*^, *tgrC1* ^*-*^ and AX4 for 20 hour, which is the peak expression time of *stcA* in AX4. We collected RNA samples and reverse transcribed the mRNA. Using the resulting cDNA, we PCR-amplified one fragment 5’ of the insertion site (A) and another fragment across the insertion site (B). Each reaction was performed with and without reverse transcriptase (RT) to control for genomic DNA contamination. The reactions were loaded side by side where “+” indicates reactions with RT, and “-” indicates reactions without RT. The relevant genotypes are indicated above the lanes. MW – molecular weight marker (base pairs). The green boxes below the gene models represent the target RT-PCR regions in each experiment. The dotted lines represent the start and the end of the RT-PCR products. The blue line represents the insertion site.
(TIF)
