# Supplementary figures and images for: TgrC1 Has Distinct Functions in Dictyostelium Development and Allorecognition
Source: PLoS One. 2015 Apr 20;10(4):e0124270. doi: 10.1371/journal.pone.0124270 (PMC4404348; doi:10.1371/journal.pone.0124270)

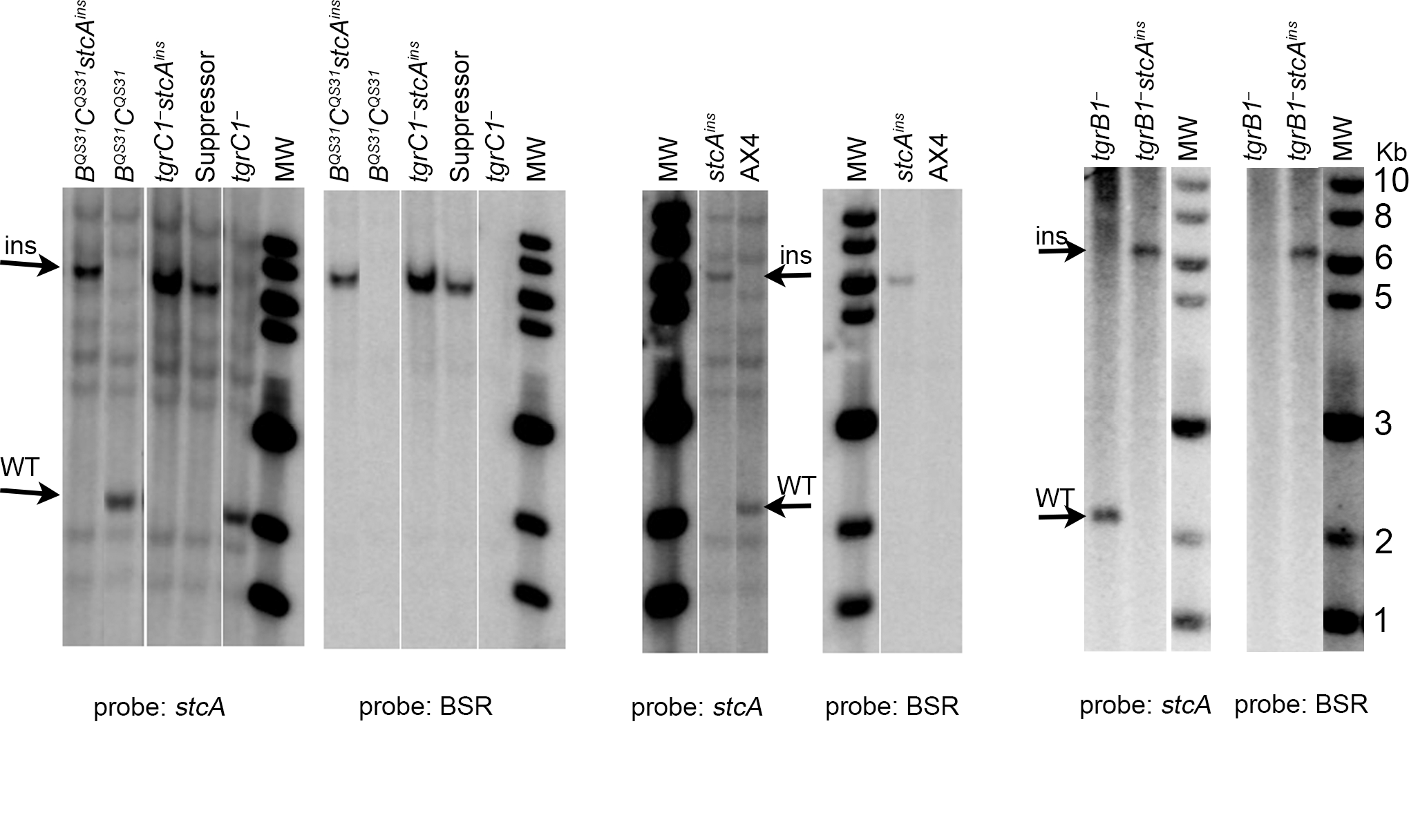

Supplement: S1 Fig — We prepared genomic DNAs from all the strains tested (relevant genotypes are indicated above the lanes; ‘suppressor’ represents the original suppressor strain), digested the DNA samples with SpeI, and separated the fragments on 0.8% agarose gel. After transferring the DNAs to nitrocellulous membranes, we processed the blots with probes against stcA or against the blasticidin S resistance cassette (BSR) as indicated below the lanes. MW – molecular weight markers (Kb). Arrows point to the wild type (WT) and mutant (ins) stcA bands. The SpeI fragment of stcA gene is about 2.2 Kb, and the pBSR1 insertion size is approximately 4.1 Kb. (TIF) [file pone.0124270.s001.tif]

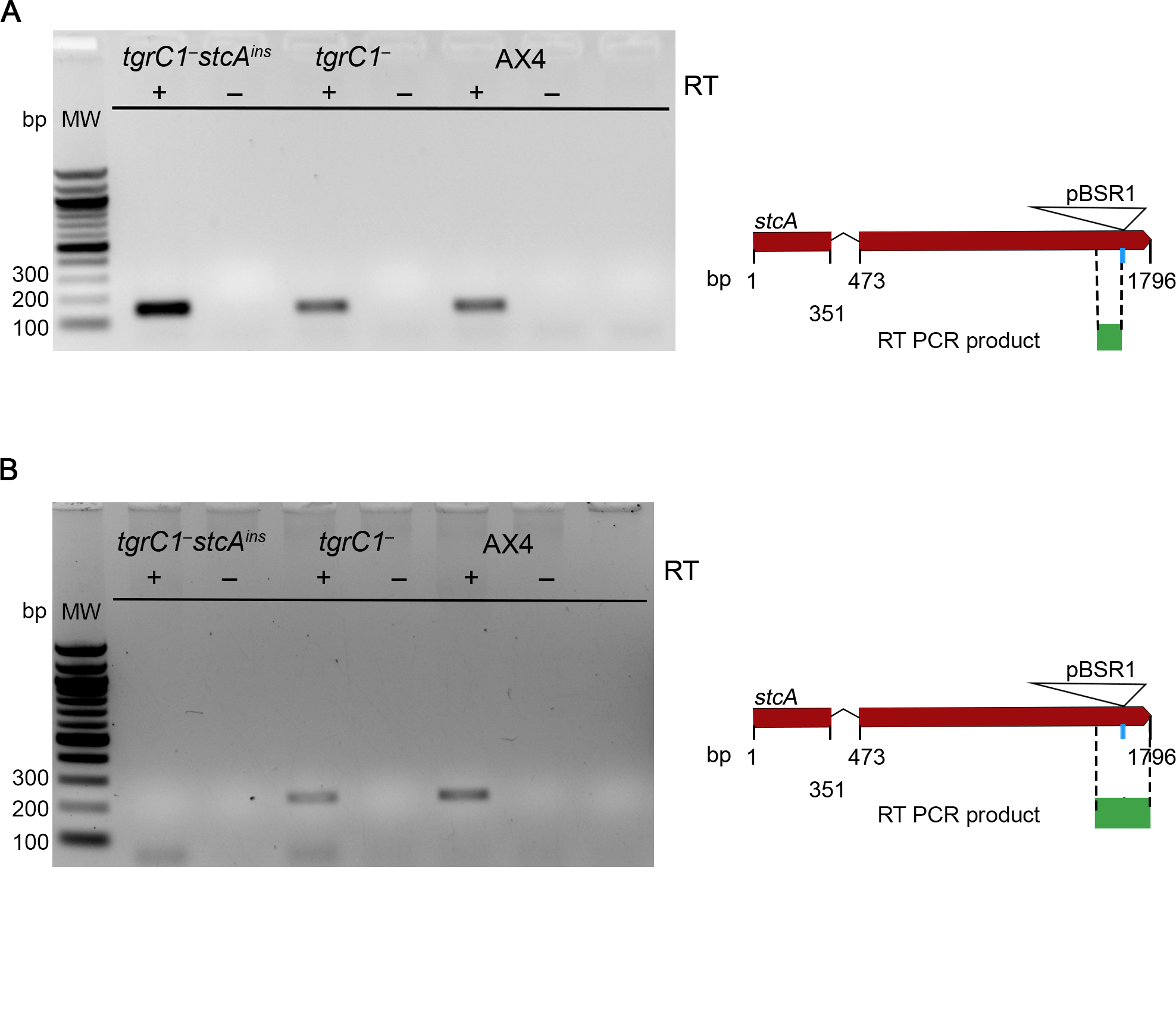

Supplement: S2 Fig — We developed the strains tgrC1 - stcA ins, tgrC1 - and AX4 for 20 hour, which is the peak expression time of stcA in AX4. We collected RNA samples and reverse transcribed the mRNA. Using the resulting cDNA, we PCR-amplified one fragment 5’ of the insertion site (A) and another fragment across the insertion site (B). Each reaction was performed with and without reverse transcriptase (RT) to control for genomic DNA contamination. The reactions were loaded side by side where “+” indicates reactions with RT, and “-” indicates reactions without RT. The relevant genotypes are indicated above the lanes. MW – molecular weight marker (base pairs). The green boxes below the gene models represent the target RT-PCR regions in each experiment. The dotted lines represent the start and the end of the RT-PCR products. The blue line represents the insertion site. (TIF) [file pone.0124270.s002.tif]
